# Interaction between Hemin and Prion Peptides: Binding, Oxidative Reactivity and Aggregation

**DOI:** 10.3390/ijms21207553

**Published:** 2020-10-13

**Authors:** Simone Dell’Acqua, Elisa Massardi, Enrico Monzani, Giuseppe Di Natale, Enrico Rizzarelli, Luigi Casella

**Affiliations:** 1 Dipartimento di Chimica, Università di Pavia, Via Taramelli 12, 27100 Pavia, Italy; elisamassardi@gmail.com (E.M.); enrico.monzani@unipv.it (E.M.); 2 Istituto di Cristallografia, s.s. Catania, Consiglio Nazionale delle Ricerche, via Paolo Gaifami 18, 95126 Catania, Italy; peppedinatale@yahoo.it (G.D.N.); erizza@unict.it (E.R.)

**Keywords:** hemin, prion peptides, prion diseases, oxidative stress, neurodegeneration, peroxidase

## Abstract

We investigate the interaction of hemin with four fragments of prion protein (PrP) containing from one to four histidines (PrP_106–114_, PrP_95–114_, PrP_84–114_, PrP_76–114_) for its potential relevance to prion diseases and possibly traumatic brain injury. The binding properties of hemin-PrP complexes have been evaluated by UV–visible spectrophotometric titration. PrP peptides form a 1:1 adduct with hemin with affinity that increases with the number of histidines and length of the peptide; the following log K_1_ binding constants have been calculated: 6.48 for PrP_76–114_, 6.1 for PrP_84–114_, 4.80 for PrP_95–114_, whereas for PrP_106–114_, the interaction is too weak to allow a reliable binding constant calculation. These constants are similar to that of amyloid-β (Aβ) for hemin, and similarly to hemin-Aβ, PrP peptides tend to form a six-coordinated low-spin complex. However, the concomitant aggregation of PrP induced by hemin prevents calculation of the K_2_ binding constant. The turbidimetry analysis of [hemin-PrP_76–114_] shows that, once aggregated, this complex is scarcely soluble and undergoes precipitation. Finally, a detailed study of the peroxidase-like activity of [hemin-(PrP)] shows a moderate increase of the reactivity with respect to free hemin, but considering the activity over long time, as for neurodegenerative pathologies, it might contribute to neuronal oxidative stress.

## 1. Introduction

Iron protoporphyrin IX is a macrocyclic complex in which iron can be coordinated in both +2 and +3 oxidation states: the iron (III) complex is defined hemin, whereas the iron (II) complex is defined heme. Heme/hemin are essential for many physiological processes, as they act as prosthetic groups of proteins and enzymes involved in oxygen transport, electron transfer, or enzymatic reactions that require oxygen or peroxide activation [1,2]. Moreover, heme has also an important role as intracellular messenger [3]. On the other hand, accumulation of free heme, generally in the oxidized hemin form, has toxic effect, due to its capacity to induce the production of reactive oxygen species (ROS) through Fenton chemistry. Therefore, several control mechanisms are active for maintaining low concentration of free hemin [4,5,6]. Moreover, hemin has also an emerging role in the regulation of several cellular processes in neurons, and therefore, hemin metabolism dysfunctions might contribute to neurodegenerative disorders [7,8,9]. For instance, the interaction between hemin and amyloid-β (Aβ) has been found to be relevant for Alzheimer’s disease [10,11,12,13,14]. 

Among neurodegenerative diseases, prion (PRoteinaceous Infective ONly particle) pathologies affect both animals (bovine or cervid spongiform encephalopathy or sheep scrapie) and man (sporadic Creutzfeldt–Jakob Desease (sCJD)). In particular, cervid spongiform encephalopathies are related to the recent and dangerous spread of Chronic Wasting Disease [15,16]. The conversion of physiological cellular prion protein (PrP^C^) into an anomalous isoform, scrapie prion protein (PrP^Sc^), is responsible for both the inception of the disease and the infectious nature of the pathogenic agent [17,18]. At the basis of this conversion, there is a change in the secondary protein structure from an α-helical conformation to a structure rich in β-sheet in the pathogenic isoform.

Prions are extracellular proteins expressed in various types of tissues but particularly abundant in the synaptic membranes of neuronal cells [19]. Prions are anchored to the outer leaflet of the membrane by a glycosyl phosphoinositoyl group [20]. The physiologic function of these proteins remains poorly understood; the current hypothesis proposes that prions modulate several synaptic mechanisms through the interaction with different proteins and receptors present at the synapses. PrP^C^ may regulate the function of its target protein by promoting post-translational modifications, for example, by inducing the S-nitrosylation of *N*-methyl-D-aspartate (NMDA) receptor [21]. This function is closely related to the ability of PrP^C^ to bind divalent cations such as zinc (II) or copper (II), and this interaction seems to be important for neuron protection [22,23]. The N-terminal domain of PrP contains six histidine residues that are able to bind several copper (II) ions depending on copper concentration, with affinity in the nanomolar range [24,25]. In particular, four histidines are present in four highly conserved octa-repeats (OR) within its N-terminal domain in the portion between residues 57 and 90 and can form complexes ranging from OR-Cu^2+^ to OR-(Cu^2+^)_4_ [26] where the metal is bound with very different affinity. Moreover, the N-terminal amino group [24] and the region between 90–114, that contains two histidines (His111 and His96) [27] can bind copper (II) ions with high affinity. In particular, copper (II) binding affinity follows the order His111 > His96 >> His77 ≈ His85 [27].

Another important aspect is that this portion of PrP^C^ is also able to bind Aβ [28], which suggests that PrP^C^ acts as a receptor that mediates the neurotoxic effect of Aβ oligomers [29,30,31]. These features lead to the hypothesis that a ternary complex copper-Aβ-prion is at the basis of different neurodegenerative diseases [32,33].

There is also evidence that hemin can interact with prion proteins [34,35,36], and the relevance of this interaction has been recently confirmed [37]. Moreover, biomimetic porphyrins and phthalocyanines containing iron (III) inhibit the conversion of cellular prion protein into the abnormal isoform in vitro, acting as potential protectors in transmissible spongiform encephalopathies [38]. Based on these observations, the possibility that hemin is a natural binding agent for PrP^C^ and acts as a neuro-protector in pathological conditions such as sCJD and cerebral hemorrhage, characterized by a significant increase concentration of free hemin, has been postulated [37]. 

Lee et al. have shown that hemin interacts with prion proteins, and this interaction might induce rapid endocytosis from plasma membrane to form endocytic compartments, even if the mechanism is still to be clarified [35]. This study also shows an increase in peroxidase reactivity of free hemin because of the coordination of a histidine of PrP^C^ to iron (III) [35]. However, these data are qualitative and neither the kinetic parameters nor the binding constants of the hemin-prion complexes have been determined.

In this study, we clarify the interaction between hemin and four fragments of N-terminal domain of PrP (PrP_106–114_, Ac-KTNMKHMAG-NH_2_; PrP_95–114_, Ac-THSQWNKPSKPKTNMKHMAG-NH_2_; PrP_84–114_, Ac-PHGGGWGQGGGTHSQWNKPSKPKTNMKHMAG-NH_2_; and PrP_76–114_, Ac-PHGGGWGQPHGGWGQGGGTHSQWNKPSKPKTNMKHMAG-NH_2_) [39] that contain from one to four histidine residues. Our goal is to determine the binding constants of hemin-PrP complexes in order to understand the conditions that enable this interaction and to establish whether it can exist in physiological and/or pathological conditions. Another important aim of the present study is to determine how the binding to PrP affects the capability of hemin to promote oxidative reactions. In this view, we perform a quantitative study on the peroxidase-like reactivity of hemin-PrP complexes. Finally, we evaluate how this interaction can induce the aggregation process of PrP fragments. 

## 2. Results and Discussion

### 2.1. Binding and Equilibria of Hemin-PrP Complexes

The analysis of binding properties of hemin with PrP fragments was carried out through spectrophotometric titration of hemin (≈3 μM) with solutions of PrP peptide, in a thermostated cell at 25 °C, in 20 mM phosphate buffer at pH 7.4, monitoring changes in the Soret band (or γ band) of the porphyrin chromophore of iron (III)-protoporphyrin IX complex. The coordinative environment and the oxidation state of the metal affect this band, which exhibits an absorption maximum at 390 nm for free hemin. 

**Figure 1:**
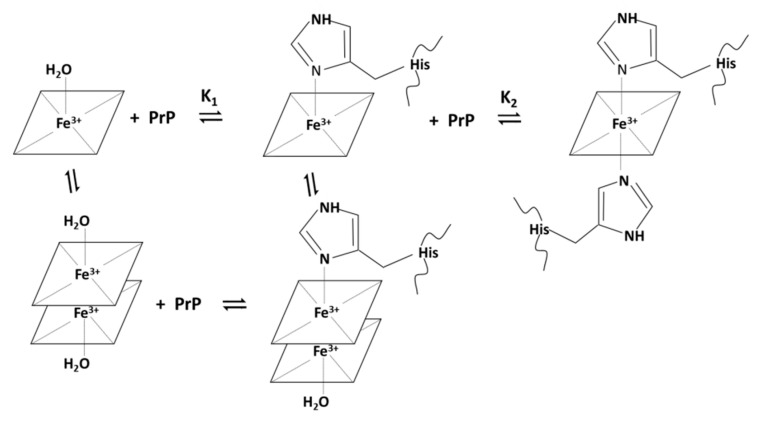
Binding equilibria of PrP histidine-containing peptides to monomeric and dimeric hemin with possible formation of five-coordinated high-spin hemin complex (equilibrium ruled by K_1_) or six-coordinated low-spin hemin:PrP complex (equilibrium ruled by K_2_).

In aqueous solution at neutral pH, hemin is present in a mixture of monomeric and dimeric species [40,41]. The addition of increasing amount of histidine-containing peptides leads to the formation of a five-coordinated high-spin complex, through an equilibrium ruled by K_1_ constant, or at higher peptide concentration, a six-coordinated low-spin complex, through an equilibrium ruled by K_2_ constant, as represented in Figure 1. The formation of the 1:1 complex only slightly affects the absorption maximum at 390 nm of free hemin, but the binding is proven by a hypochromic effect. The formation of 1:2 low-spin complex is accompanied by more marked changes, with a red shift of Soret band to 414 nm that also becomes sharper. 

#### 2.1.1. Spectrophotometric Titration of Hemin with PrP_106–114_

By adding increasing amounts of PrP_106–114_ to an aqueous hemin solution at pH 7.4, only a small decrease of the Soret band is noted, even at relatively large excess of peptide (100 equivalents) (Figure S1). This behavior indicates that hemin has low affinity for PrP_106–114_. A 1:1 high-spin species is formed, but the single histidine present in this peptide does not warrant a strong coordination to stabilize the complex. Therefore, this weak affinity interaction bears potentially low physiological importance.

#### 2.1.2. Spectrophotometric Titration of Hemin with PrP_95–114_

**Figure 2:**
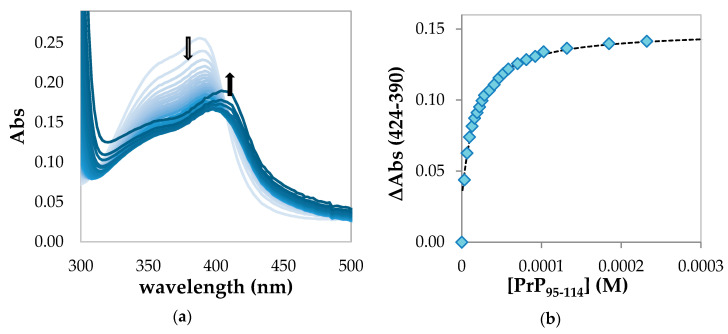
(**a**) UV–Vis spectrophotometric titration in 20 mM phosphate buffer solution, pH 7.4, showing the decrease in intensity of the Soret band upon binding of hemin (3.25 µM) with PrP_95–114_ (from 0 to 90 equiv., light blue to blue) in a cell of 1 cm path length. The arrow pointing down at 390 nm indicates the hypochromic effect due to the formation of a high-spin hemin-PrP complex; the arrow pointing up at 416 nm indicates the incipient formation of a low-spin complex; (**b**) absorbance changes with respect to free hemin at 424 nm with subtraction of the contribution at 390 nm vs. PrP_95–114_ concentration fitted by high-affinity equation described in the Materials and Methods section.

In the first part of the titration of hemin with PrP_95–114_, a significant hypochromic effect of the absorption band at 390 nm is observed (Figure 2a). This behavior is attributable to the formation of a high-spin complex with a 1:1 stoichiometry between hemin and PrP_95–114_, in which iron (III) is coordinated with a histidine residue.

At larger peptide excess, absorption spectra show an increasing contribution by a band with maximum above 400 nm, which indicates the partial formation of a low-spin 1:2 hemin:peptide complex. However, the increase of the baseline over 450 nm and the absence of a clean isosbestic point indicate that a precipitation process is occurring. 

The affinity constant for the 1:1 adduct can be determined by fitting the variations of absorbance between 390 and 424 nm with the equation described in the Materials and Methods section (Figure 2b). From the average of the fitting of a different dataset (Figure S2), we obtained the following parameters: K_1_ = (6.3 ± 0.6) × 10^4^ M^−1^ and Log K_1_ = 4.80 ± 0.04.

It is important to remark that K_1_ values obtained by this method for this complex and for those obtained in the presence of PrP_84–114_ and PrP_76–114_ peptides (see Section 2.1.3 and Section 2.1.4) are affected by significant standard deviation due to the necessity to use the high affinity equation for the fitting of the experimental data. To reduce the uncertainty in K_1_ values, the binding experiment should be performed with much lower hemin concentrations. However, this would alter the hemin aggregation state (the ratio between monomer and dimer), and the binding data would not be comparable with those obtained at higher concentration for the other hemin-peptides (see comparison at Section 2.1.5). Moreover, it should be noted that the same, relatively high (3 µM), hemin concentration was used in both the binding and kinetic experiments to assess the peroxidase-like activity of the complexes described in Section 2.3. As described below, a lower hemin concentration would be not suitable for the catalysis studies due to the relatively low activity of the complexes.

#### 2.1.3. Spectrophotometric Titration of Hemin with PrP_84–114_

**Figure 3:**
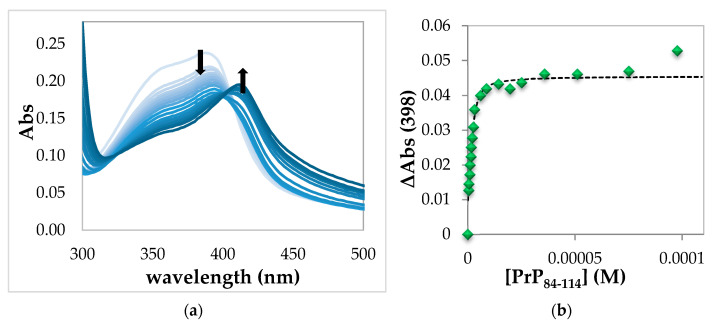
(**a**) UV–Vis spectrophotometric titrations in 20 mM phosphate buffer solution, pH 7.4, showing the decrease in intensity of the Soret band upon binding of hemin (2.80 µM) with PrP_84–114_ (from 0 to 40 equiv., light blue to blue) in a cell of 1 cm path length. The arrow pointing down at 390 nm indicates the hypochromic effect due to the formation of a high-spin hemin-PrP complex; the arrow pointing up at 416 nm indicates the incipient formation of a low-spin complex; (**b**) absorbance changes with respect to free hemin at 398 nm vs. PrP_84–114_ concentration fitted by high-affinity equation described in the Materials and Methods section (the sign of ΔAbs (398) has been changed in order to obtain positive values).

The absorption spectra obtained from the titration of hemin with PrP_84–114_ show an initial decrease of the hemin Soret band at 390 nm and a subsequent increase of a band at 410 nm at higher peptide concentration (Figure 3a). 

**Figure 4:**
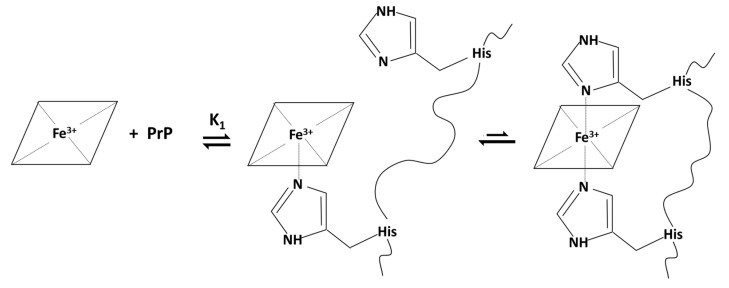
Schematic representation of the formation of 1:1 complex with possible equilibrium between five- and six-coordinated species. The peptide chain of a single peptide can coordinate hemin with two histidine residues at the same time; in the 1:1 complex five- and six-coordinated species are in equilibrium.

As in the case of PrP_95–114_, the 1:1 complex is formed in the first part of the titration, but with excess of peptide, the formation of 1:2 hemin:PrP complex is more evident than the previous case. Again, the absence of an evident isosbestic point and the increase of the absorbance over 450 nm indicate that some precipitation is occurring. However, the formation of 1:1 complex and the incipient formation of 1:2 adduct can be explained by the following two hypotheses. In the first one, illustrated in the diagram below (Figure 4), after the formation of the 1:1 complex, the peptide chain bends to coordinate hemin with two His residues belonging to the same peptide chain, forming the low spin complex. The 1:1 five- and six-coordinated forms are in equilibrium. 

**Figure 5:**
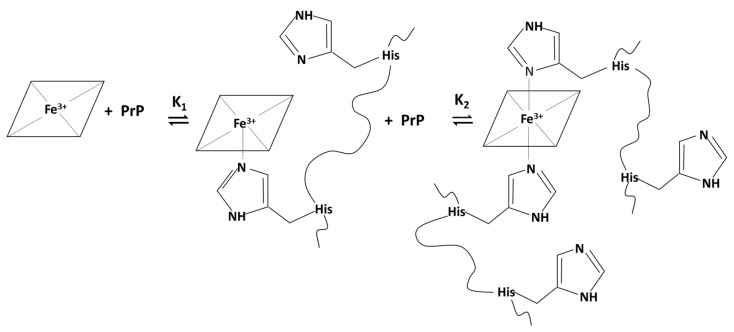
Schematic representation of the formation of 1:1 high-spin five-coordinated and 1:2 low-spin six-coordinated complexes through two distinct subsequent equilibria, ruled by K_1_ and K_2_ affinity constants, respectively.

The second hypothesis is that there are two different peptide binding equilibria to form the five- and the six-coordinated complexes (Figure 5). However, the six-coordinated 1:2 species is not completely formed at the end of the titration because of the incipient precipitation. 

The validation of these two hypotheses can be made by analyzing the binding curve obtained from the difference between the spectral variation a 390 and 424 nm against [PrP_84–114_] (Figure S3). If only one peptide binding equilibrium were present, according to the first hypothesis, a single hyperbolic curve would be expected. However, the curve shows two different phases, indicating that in solution there are two equilibria corresponding to the formation of the 1:1 and 1:2 complexes between hemin and peptide as suggested by the second hypothesis. It is therefore possible to fit the experimental data by the two-step equation described in the Materials and Methods section and obtain the binding constants for the consecutive equilibria, K_1_ = (3.1 ± 0.9) × 10^6^ M^−1^ and K_2_ = (5.2 ± 0.8) × 10^4^ M^−1^. 

It is worth noting that the constants obtained with this equation are approximated because the equation is strictly valid only for low affinity two steps equilibria, and the formation of the six-coordinated low-spin species is incomplete due to precipitation. It is therefore possible to select different wavelengths in order to selectively analyze the two equilibria and obtain more accurate values. The formation of low-spin complex in the second equilibrium is conveniently followed at 424 nm, although the observation is strongly disturbed by precipitation (data not shown). Moreover, the high peptide concentrations required to obtain the 1:2 species makes this interaction not fully relevant from the physiological point of view. On the other hand, it is of considerable interest to obtain an accurate value for the equilibrium constants of the high spin 1:1 species. 

In order to minimize the contribution of the six-coordinated species, it is possible to follow the absorbance changes in proximity of the isosbestic point at 398 nm (Figure 3b). The binding curve obtained can be fitted by a high-affinity equation described in the Materials and Methods section. The binding constant obtained for the 1:1 five-coordinated complex hemin-PrP_84–114_ is K_1_ = (1.3 ± 0.9) × 10^6^ M^−1^, Log K_1_ = 6.1 ± 0.3. These values are resulting from the average of the fitting of a different dataset (Figure S4). Notably, this value of K_1_ is in the same order of magnitude to K_1_ obtained with the two-step equation described above. 

#### 2.1.4. Spectrophotometric Titration of Hemin with PrP_76–114_

**Figure 6:**
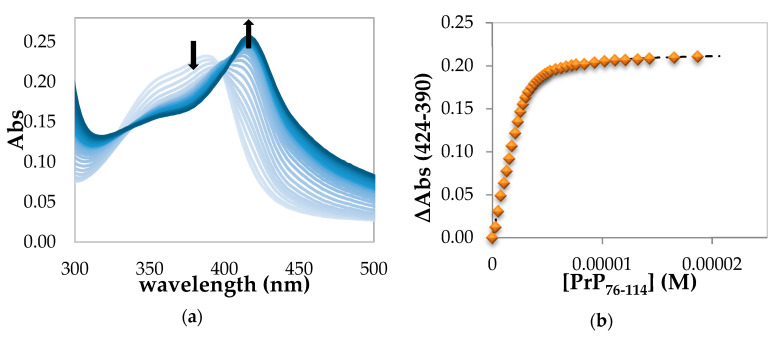
(**a**) UV–Vis spectrophotometric titrations in 20 mM phosphate buffer solution, pH 7.4, showing the decrease in intensity of the Soret band upon binding of hemin (2.74 µM) with PrP_76–114_ (from 0 to 7 equiv., light blue to blue) in a cell of 1 cm path length. The arrow pointing down at 390 nm indicates the hypochromic effect due to the formation of a high-spin hemin-PrP complex; the arrow pointing up at 416 nm indicates the formation of a low-spin complex; (**b**) absorbance changes with respect of free hemin at 424 nm with subtraction of the contribution at 390 nm vs. PrP_76–114_ concentration fitted by high-affinity equation described in the Materials and Methods section.

The absorption spectra obtained upon titration of hemin with PrP_76–114_ show a red shift of the Soret band even with addition of sub stoichiometric amounts of peptide (Figure 6a).

In this case, the low-spin complex characterized by Soret band at 414 nm is completely formed with less than 2 equivalents of PrP_76–114_. Then we assume that for this peptide the binding equilibrium involves 1:1 stoichiometry, as shown in Figure 4, with the intra-molecular His binding equilibrium shifted toward the six-coordinated, low-spin, species. However, the absence of a clear isosbestic point and the increase of baseline indicate that also in this case a precipitation process is occurring. In this hemin-PrP_76–114_ complex, the peptide fragment coordinates the iron (III) center using two histidine residues of the same backbone. An estimate of the binding constant of this interaction was obtained from the absorbance change at 424 nm upon subtraction of the contribution at 390 nm. The plot obtained (Figure 6b) was fitted with a one-step binding equation that allows to calculate K_1_ = (3.0 ± 0.9) × 10^6^ M^−1^, Log K_1_ = 6.48 ± 0.25. These values result from the average of the fitting of a different dataset (Figure S5). This case is different from the situation observed for PrP_95–114_ and PrP_84–114_ peptides where the K_1_ values are referred to 1:1 high-spin complex that can be converted to a 1:2 adduct upon increase of the excess of PrP peptide. The length and the multiple histidine of PrP_76–114_ allow the saturation of iron coordination to form a stable 1:1 low-spin complex. Moreover, the presence of a higher number of proline residues might favor the folding of peptide fragments and, therefore, the formation of macrochelates.

#### 2.1.5. Comparison between Binding Constants of the Complexes between PrP Peptides and Other Neuronal Peptides

With the exception of PrP_106–114_, all PrP peptides form a 1:1 iron (III)-PrP complex and the corresponding formation constant (K_1_) could be determined. In the presence of excess PrP_95–114_ and PrP_84–114_ the low-spin 1:2 iron (III)-PrP complex can be formed although the relative binding constant could not be determined due to precipitation. Only for hemin-PrP_84–114_ complex, an estimate of the K_2_ value was possible. As described above, the K_1_ constant obtained in the presence of PrP_76–114_, K_1_ is referred to the 1:1 in which two histidine of the same backbone are coordinated to hemin. In PrP_76–114_, the possible isomers of the ternary complexes could explain the different behavior between the PrP_84–114_and PrP_76–114_. From the comparison of K_1_ values summarized in Table 1, it is clear that the binding constant for the hemin-PrP 1:1 complex increases with the number of histidine residues in the sequence (highest for PrP_76–114_). Moreover, peptides that contain the histidine residues included in the OR region (PrP_84–114_ and PrP_76–114_) display the larger affinity values. Notably, the binding constant of hemin complexes with PrP_84–114_ and PrP_76–114_ peptides are two orders of magnitude larger than that of hemin-PrP_95–114_ complex.

The binding constants obtained for hemin-PrP peptides are compared in Table 1 with those reported for other hemin complexes with neuronal peptides, in particular Aβ [42,44,45] and a fragment of tau protein [43]. The K_1_ binding constant of hemin with PrP_84–114_ and PrP_76–114_ is similar to that hemin-AcR1τ and hemin-Aβ42 complexes, whereas the binding constant of hemin-PrP_95–114_ is similar to that of hemin-Aβ16. From these data, we can conclude that the length of the peptide is important, since longer peptides as PrP_84–114_, PrP_76–114_ and Aβ42 form stronger complexes with hemin compared to PrP_95–114_ and Aβ16. As for Aβ peptides, PrP fragments display the capability to form low-spin six-coordinate complexes with hemin. Again, it is important to highlight that the peculiar behavior of PrP_76–114_, also compared with Aβ peptides, because it is the only case that presents a 1:1 low-spin complex. The remarkable tendency of hemin-PrP complexes to form aggregates prevents the calculation of reliable K_2_ binding constants, with the exception of hemin-PrP_84–114_ complex. For this reason, as described below, we performed an analysis of the aggregation process through turbidimetry assay. 

### 2.2. PrP Aggregation Induced by Hemin Studied by Turbidimetry Assay

The aggregation process and the formation of amyloid fibrils of prion protein is the key event associated with the onset of prion diseases. The portion between the residues 90–140 has been historically identified as the most important for the aggregation process (amyloidogenic region), even if recently the hypothesis that also the region from 170 to 220, including helix 2 and helix 3, has been raised [46,47]. 

The PrP peptides used in this study include a portion of the amyloidogenic region, and it is therefore important to establish if the interaction with hemin affects the tendency of prion peptides to aggregate. The aim of the study was to investigate the aggregation propensity of PrP_76–114_ and PrP_84–114_, in the presence of hemin by monitoring the turbidity of the solution at 405 and 750 nm over time, in PBS buffer at pH 7.4 with hemin:PrP 1:2 stoichiometry (8 μM peptide and 4 μM hemin), at room temperature. 

As observed in the spectrophotometric titration, hemin and PrP_76–114_ at 1:2 stoichiometry forms a six-coordinated complex with an incipient precipitation (Figure S6, panel (a), light blue spectrum). By monitoring the visible spectra over time, we observe a general decrease of the absorption at 405 nm that indicates a precipitation process (Figure S6, panel (b), black trace). At 750 nm (Figure S6, panel (b), red trace), there is an increase of absorbance, which ends within 50 min of reaction, that indicates an aggregation process; after this time, the absorbance is stable probably because the increase of turbidimetry is compensated by the precipitation process. Notably, the precipitation process occurs in a fast time scale since more than 50% of hemin precipitates over 14 h reaction time. 

This behavior is widely different if compared with the previous study on the interaction of hemin with R1τ and AcR1τ peptides, in which an increase of absorbance at 405 and 750 nm indicates an increase of turbidimetry and, therefore, an aggregation process, over a reaction time of 7 days [41].

This assay in the presence of PrP_84–114_ shows again an increase of absorbance at 750 nm which expires within 50 min of reaction (Figure S7, panel (b), red trace), followed by a steady kinetic profile. In addition, the almost stable absorbance at 405 nm indicates that once aggregated the hemin-PrP_84–114_ complex is less prone to precipitation compared to PrP_76–114_.

In conclusion, the presence of hemin induces a rapid precipitation of the longest peptide of prion protein, PrP_76–114_, confirming that this interaction drastically reduces the PrP solubility, especially considering that in the presence of the full protein this situation might be further exacerbated. These data corroborate a previous study that shows that the presence of copper and zinc increases the PrP tendency to aggregate [48]. Finally, the characterization of the precipitated peptide clearly deserves more detailed consideration.

### 2.3. Hydrogen Peroxide Activation

The binding of neuronal proteins to transition metal ions, and to the heme group, has the important consequence of promoting redox reactions in the resulting complexes. This is particularly relevant for the oxidative reactions that can be harmful for the biological environment [49,50]. To gain an appreciation of the catalytic oxidative potential of hemin-PrP complexes, we compare the pseudo-peroxidase activity of free hemin with that of hemin-PrP complexes against the standard substrate 2,2′–azino-bis(3-ethylbenzothiazoline-6-sulfonic acid) (ABTS). The kinetic analyses of the peroxidase-like reactions were performed in phosphate buffer at pH 7.4 using hydrogen peroxide as oxidant. 

The oxidation processes promoted by hemin complexes can be described with reference to a catalytic mechanism similar to that of peroxidases and microperoxidases [51,52,53,54], which can be summarized in the following three steps:

PFe^III^ + H_2_O_2_ → ^•+^PFe^IV^ = O + H_2_O (1)

^•+^PFe^IV^ = O + SH ⇄ [^•+^PFe^IV^ = O/SH] → PFe^IV^ = O + S^•^ + H^+^ (2)

PFe^IV^ = O + SH ⇄ [PFe^IV^ = O/SH] + H^+^ → PFe^III^ + S^•^ + H_2_O (3)

where PFe^III^ indicates the iron porphyrin in its resting state, ^•+^PFe^IV^ = O the first catalytic intermediate (resembling Compound I in peroxidases), PFe^IV^ = O the second catalytic intermediate (corresponding to Compound II), and SH a generic substrate. The rate determining step of the process depends on the H_2_O_2_ and substrate concentrations, but usually, it corresponds to reaction 1 or reaction 3, since the ^•+^PFe^IV^ = O species is the most reactive intermediate. The rate of reaction 1 is controlled by the second order rate constant *k*_1_, whereas that of reaction 3 by the constants K_M_ (generally assumed as the dissociation constant of the [PFe^IV^ = O/SH] adduct) and *k*_cat_, the maximum turnover rate of the catalyst.

For each hemin-PrP complex, we performed an initial analysis to determine the peptide concentration maximizing the peroxidase activity, taking into account that a free coordination position on iron in the 1:1 five-coordinated hemin-peptide complex is required to react with peroxide. The experiments were carried out in conditions of saturating peroxide and ABTS concentrations, at fixed concentration of hemin (3 μM) and varying peptide concentration. The peptide concentration thereby obtained is then used in subsequent studies in which hydrogen peroxide or substrate concentration are varied, respectively. 

After optimization of the peptide concentration, we investigated the peroxidase-like reactivity under conditions in which the rates do not depend on substrate concentration but are linearly dependent on hydrogen peroxide concentration [52,53]. This means that the rate-limiting step of the reaction is the formation of the high valent ^+•^PFe^IV^ = O complex (reaction 1), with rate constant *k*_1_, while the reaction with the substrate occurs in a fast step. The *k*_1_ rate constants for the series of hemin complexes were determined from the slope of the rate vs. [H_2_O_2_] plots (Figure S8); the data are reported in Table 2. To neglect the competitive degradation undergone by the hemin catalysts during the reactions, particularly at (relatively) high H_2_O_2_ concentration, the reaction rates were calculated from the initial phase of the reaction.

For the determination of the kinetic parameters for reaction 3, the rates were evaluated by varying the substrate concentration at fixed, and saturating, hydrogen peroxide concentration. The reaction rates follow a hyperbolic behavior (Figure S9), which could be interpolated with Michaelis–Menten equation, obtaining the K_M_, *k*_cat_ and *k*_cat_/K_m_ parameters reported in Table 2. 

These data show that the peroxidase activity of the hemin-PrP complexes is slightly higher than that of free hemin, indicating that in physiological conditions this reactivity should not be harmful, at least in the short term. The modest peroxidase reactivity is mainly due to the low rate of formation of the high-valent ^•^PFe^IV^ = O intermediate, in the first step of the catalytic cycle (reaction 1, ruled by *k*_1_), but also to the low catalytic efficiency in terms of turnover rate (*k*_cat_). Regarding the rate of formation of the first catalytic intermediate (reaction 1), in peroxidases, it strictly depends on the effect of activation of the peroxide exerted by key distal residues. The histidine and arginine residues present in the distal pocket of the enzyme polarize the peroxide O-O bond by acting as acid-base catalysts and facilitating the breaking of the bond. In PrP peptides, as well as in Aβ and AcR1τ, positively charged (e.g., Lys) and acid-base (e.g., His) residues that could play this role are present, but the lack of secondary structure prevents a similar effect. The *k*_1_ constants of all the hemin complexes are, in fact, only slightly higher than that of free hemin, indicating that the coordination of the peptides to the metal only marginally affects peroxide activation. Statistically, (T-test, *p* = 0.05, *n* = 3) *k*_1_ of all hemin-PrP complexes (apart from hemin-PrP_106–114_ complex) is different from that of free hemin. Finally, we should note that the reduced rate observed for hemin-PrP_76–114_ is due to the presence of a second histidine coordinated to iron (III), which clearly competes with the binding of hydrogen peroxide. 

The comparison of K_M_ values shows that the hemin-PrP complex with shorter peptide fragments displays an increase in K_M_ compared to free hemin, indicating a lower affinity for ABTS, even if only K_M_ for compounds hemin-PrP_106–114_ and hemin-PrP_76–114_ complexes are significantly different from that of hemin. On the other hand, by increasing the length of PrP peptide, the K_M_ decreases, with values similar (hemin-PrP_85–114_) or smaller (hemin-PrP_76–114_) than for free hemin. This is probably due to the possibility to establish effective interactions between the peptide and the substrate, likely through the non-coordinating histidine. More significant is the increase of *k*_cat_ parameters for all hemin-peptide complexes, all statistically relevant, which in the case of PrP peptides amounts to 5–6 folds. Thus hemin binding to the peptides facilitates the electron transfer from the substrate to the PFe^IV^ = O intermediate in the slow step of the catalytic cycle, probably because the interaction with the peptide favors better positioning of the substrate close to the porphyrin, even though this effect cannot compare with the binding site present in peroxidases [55]. Moreover, the histidine coordination increases the reaction rate by diminishing the structural reorganization required for the electron transfer between the substrate and the PFe^IV^ = O intermediate, resembling the effect of proximal histidine in peroxidases. 

Although the catalytic activity of hemin-PrP and the other hemin-peptide complexes is limited, it is important to emphasize that the peptides bear a promotion effect and that this activity can be prolonged over long periods, as typically occurs in the neuroinflammatory processes accompanying neural degeneration. Therefore, also the moderate effect produced by the presence of prion peptides can induce an increase in oxidative cellular damage. The damaging effect can be considerably aggravated in cases of traumatic brain injury, where a massive release of hemoglobin, and consequently free hemin, occurs [56]. 

## 3. Materials and Methods

### 3.1. Materials and Instrumentation

Protected amino acids, rink amide resin, and other reagents for peptide synthesis, i.e., benzotriazol-l-yl-oxytripyrrolidinophosphonium hexafluorophosphate and *O*-benzotriazole-*N,N,N’,N’*-tetramethyluronium hexafluoro-phosphate, were purchased from Novabiochem (Merck KGAA, Darmstadt, Germany). All other chemicals were reagent grade from Sigma-Aldrich (Merck KGAA, Darmstadt, Germany). Peptide purifications were performed on a Jasco (JASCO International Co. Ltd., Hachioji, Tokyo, Japan) HPLC instrument equipped with two PU-1580 pumps and a MD-1510 diode array detector (working range: 195–659 nm), using a Phenomenex Jupiter 4U Proteo semi-preparative column (250×10 mm). Mass spectra were recorded using a Thermo-Finnigan (San Jose, CA, USA) LCQ ADV MAX ion-trap mass spectrometer, with an ESI ion source. UV–Vis spectra were recorded on an Agilent 8453 diode array spectrophotometer, equipped with a thermostated, magnetically stirred optical cell. 

### 3.2. Peptide Synthesis

The peptides PrP_106–114_ (Ac-KTNMKHMAG-NH_2_, mw 1057.5) [57] PrP_84–114_ (Ac-PHGGGWGQGGGTHSQWNKPSKPKTNMKHMAG-NH_2_, mw 3297.65) [58] and PrP_76–114_ (Ac-PHGGGWGQPHGGWGQGGGTHSQWNKPSKPKTNMKHMAG-NH_2_, mw 4074.45) [39] were synthesized according to literature procedures. 

PrP_95–114_ (Ac-THSQWNKPSKPKTNMKHMAG-NH_2_, mw 2350.01) was synthesized using the standard fluorenyl methoxycarbonyl (Fmoc) solid-phase synthesis in dimethylformamide (DMF). Rink-amide resin MBHA (substitution 0.78 mmol/g) was used as polymeric support, which yielded the peptide amidated at the C-terminus. After deprotection of the resin by treating the support twice, for 3 min and 7 min, with 20 mL of 20% (v:v) piperidine in DMF, the first amino acid (2 mol equiv. vs. resin sites) was added in the presence of 2 equiv. of *N*-hydroxybenzotriazole (HOBt), 2 equiv. of benzotriazol-1-yl-oxytripyrrolidinophosphonium hexafluorophosphate (PyBOP) and ≈ 2 equiv. of *N,N*-diisopropylethylamine (DIEA). After 45 min, the same coupling procedure was repeated. After recoupling of each amino acid, a capping step was performed by using 20 mL of 4.7% acetic anhydride and 4% of pyridine in DMF; resin was washed by DMF, dichloromethane and isopropanol. At the end of the synthesis, the protections of the side chains of the amino acids were removed with a solution of 95% trifluoroacetic acid (TFA, 25 mL for 1 g of resin), triisopropyl silane (2.5%) and water (2.5%), which serves also to release the peptide from the resin. After stirring for 3 h, cold diethyl ether was added to precipitate the peptide and the mixture was filtered. Then, it was dissolved in water and purified by HPLC, using a linear gradient from 80 : 20 = 0.1% TFA in water: 0.1% TFA in CH_3_OH to 50 : 50 = 0.1% TFA in water : 0.1% TFA in CH_3_OH over 40 min (flow rate of 4 mL/min, loop 2 mL), as eluent. PrP_95–114_ showed a retention time of ≈ 25 min. The product was then lyophilized and stored at −30 °C until use. The identity of the peptide was confirmed by Electrospray ionization mass spectrometry (Thermo-Finnigan). ESI-MS data (direct injection, MeOH, positive-ion mode, capillary temperature 200 °C): *m/z* 1175 (PrP_95–114_^2+^), 784 (PrP_95–114_^3+^), 588 (PrP_95–114_^4+^), 471 (PrP_95–114_^5+^).

Peptide concentration was determined via tryptophan absorbance at 281 nm with an extinction coefficient of 5690 M^−1^cm^−1^ [59]. 

### 3.3. Binding Experiments

All glassware and optical cells used for spectral measurements were carefully cleaned following recommended procedures [40,41]. A 4 mM stock hemin solution was freshly prepared by dissolving hemin *b* (iron (III)-complex) in 0.1 M NaOH. Care was taken to ensure that hemin was fully dissolved after sonication of the solutions for several minutes. Working solutions of hemin were prepared by diluting the stock solution in the buffered aqueous solvent. Concentration of hemin solution was measured by UV–vis spectroscopy using the molar extinction coefficient ε_390_ = 84,000 M^−1^cm^−1^ [60]. The equilibrium constants for ligand binding were estimated by spectrophotometric titration of solutions of hemin (about 3 µM, the exact concentration is reported for each experiment) in 20 mM phosphate buffer at pH 7.4 with solutions of PrP_106–114_, PrP_95–114_, PrP_84–114_, and PrP_76–114_.

Spectrophotometric titration experiments were performed in thermostated cells at 25 °C. All titrations were carried out after stabilization of the absorbance of the solutions of the hemins and the UV–Vis spectra were recorded after 60 s from each addition of the peptide (PrP_106–114_, PrP_95–114_, PrP_84–114_ and PrP_76–114_), under stirring. Titration data were processed with the Fig.P [61] program using a range of wavelengths centered on the Soret absorbance maximum (from 360 nm to 430 nm). The equilibrium constants were determined from the plots of absorbance changes with respect to free hemin, corrected for dilution, against added ligand equivalents. 

The K_1_ constant relative to the formation of 1:1 hemin:PrP complexes was calculated with the following equation:

(4) $$\Delta Abs=\left\{\frac{\Delta A_{\infty }}{2\left[E_{0}\right]K_{1}}\left[K_{1}\left(\left[E_{0}\right]+\left[L_{i}\right]\right)+1\;-\sqrt{K_{1}{}^{2}{\left(\left[L_{i}\right]-\left[E_{0}\right]\right)}^{2}+2K_{1}\left(\left[E_{0}\right]+\left[L_{i}\right]\right)+1}\right]\right\}+B\;$$

where ΔAbs, absorbance variation with respect to free hemin; ΔA_∞_, limiting value for ΔAbs; K_1_, affinity binding constant; E_0_, initial hemin concentration; L_i_, total (free + bound) peptide concentration at each addition; B, additional constant that considers that hemin is still predominantly aggregated at the beginning of the titration (this term was negligible for hemin-PrP_76–114_ complex formation). 

For hemin-PrP_84–114_ complex formation, the following two-step low-affinity binding equation was used for the calculation of K_1_ and K_2_ constants:

(5) $$\Delta Abs=\left\{\frac{A_{1}K_{1}x+A_{2}K_{1}K_{2}x^{2}}{1+K_{1}x+K_{1}K_{2}x^{2}}\right\}$$

where ΔAbs, absorbance variation with respect to free hemin; A_1_ and A_2_, absorbance difference with respect to free hemin for the 1:1 and 1:2 hemin:PrP complexes, respectively; K_1_, binding constant for the formation of 1:1 hemin:PrP complex; K_2_, binding constant for the formation of 1:2 hemin:PrP complex; *x*, peptide concentration after each addition. The equation has been obtained considering low-affinity bindings; when applied to high affinity binding equilibria, it gives an estimate of the constants. The binding constants obtained from the average values obtained by two separate datasets.

### 3.4. Turbidity Measurements

The PBS buffer at pH 7.4 (137 mM NaCl, 3 mM KCl, 10 mM Na_2_HPO_4_, 2 mM KH_2_PO_4_, ionic strength ~160 mM) was treated with Chelex, under stirring for 24 h. The resin was filtered through a 0.2 μm filter before use of the buffer. PrP_84–114_ or PrP_76–114_ (8 µM) and hemin (4 μM) were added in the cuvette (final volume 2 mL) and the UV–visible spectrum was monitored over 15 h. 

### 3.5. Kinetic Experiments

The catalytic activities of the hemin-peptide complexes were analyzed by following the H_2_O_2_-catalyzed oxidation of ABTS in 20 mM phosphate buffer at pH 7.4 and 25 °C, using thermostated 1-cm optical cells under magnetic stirring. The reactions were initiated by the addition of hydrogen peroxide as the last reagent and were followed through the development of the optical band of ABTS^+·^ radical at 660 nm (ε_660_ = 14,700 M^−1^ cm^−1^) [62]. Reaction rates in Δabsorbance/s units were calculated from the slope of the trace in the initial 30 s, but discarding the first few seconds, to allow stabilization of the readings, and were then converted in turnover rates (s^−1^) by dividing by the catalyst concentration, the molar extinction coefficient of ABTS^+^ and a factor of 2 because each catalytic cycle involves formation of two ABTS^+^ molecules. 

We preliminarily evaluated the rate dependence on PrP peptide concentration to determine the conditions in which maximum reactivity is achieved. In this study, the following conditions were used: 3 µM hemin, 1 mM ABTS, 10 mM H_2_O_2_ and increasing concentration of PrP peptides. The maximum reactivity was reached at the following PrP peptide concentrations: 90 µM PrP_106–114_, 4.8 µM PrP_95–114_, 6 µM PrP_84–114_, and 3.6 µM PrP_76–114_.

The rate dependence on H_2_O_2_ concentration was studied by reacting each of the hemin-peptide complexes (3 µM hemin, and 90 µM PrP_106–114_, 4.8 µM PrP_95–114_, 6 µM PrP_84–114_, and 3.6 µM PrP_76–114_) with ABTS (1 mM), and hydrogen peroxide (from 50 µM to 20 mM). The *k*_1_ values were obtained from the slope of the linear part, at low peroxide concentrations, of the rate/[catalyst] vs. [H_2_O_2_] graphs. 

The rate dependence on ABTS concentration was studied by keeping constant the catalyst (3 µM hemin, and 90 µM PrP_106–114_, 4.8 µM PrP_95–114_, 6 µM PrP_84–114_, and 3.6 µM PrP_76–114_) and hydrogen peroxide (5 mM for hemin, and 11 mM PrP_106–114_, 10 mM PrP_95–114_, 8 mM PrP_84–114_, and 8 mM PrP_76–114_) concentrations, while that of ABTS was changed from 100 µM to 15 mM). In all cases, the optical traces at 660 nm displayed a hyperbolic behavior and could be fitted with the Michaelis–Menten equation. The difference between the kinetic parameters obtained with hemin-PrP complexes compared to that of free hemin were tested by T-test (*p* = 0.05, *n* = 3). Control experiments showed that the absorbance changes in the absence of H_2_O_2_ or the hemin catalysts were completely negligible. 

## 4. Conclusions

We have investigated in detail the binding and reactivity of ferric heme with four fragments of different length of the N-terminal portion of prion protein, which contain from one (PrP_106–114_) up to four (PrP_76–114_) histidine residues. This region is involved in the aggregation process of PrP but is also important for metal binding (mainly divalent ions as copper (II) and zinc (II), and for the interaction with Aβ peptide. 

The interaction between hemin and PrP has been already proposed to be physiologically relevant [33,34,35] and, therefore, a main purpose of the present study was to determine the extent and the magnitude of the binding. The peptide length and number of histidine residues strongly affect the mode of binding and the strength of association. In particular, all the peptides form 1:1 five-coordinated high-spin complexes with the exception of PrP_76–114_ that forms a 1:1 six-coordinated low-spin adduct. In the presence of an excess of PrP_95–114_ and PrP_84–114_ peptides, the formation of a 1:2 six-coordinated low-spin complex is observed. The binding constants (K_1_) referred to the formation of 1:1 species show that the complex is strongest with the longest peptide, PrP_76–114_, containing four histidine residues, and diminishes with the length of the peptide chain, with PrP_106–114_ displaying a weak interaction. The K_1_ values obtained for PrP_76–114_, PrP_84–114_ and PrP_95–114_ are comparable with those reported for other important neuronal peptides such as Ab42, Ab16, and AcR1τ. 

The formation of 1:2 six-coordinated low-spin complexes has been observed for PrP_84–114_ and PrP_95–114_, but the aggregation process occurring concomitantly prevents the calculation of a K_2_ binding constant. 

These aspects could indicate that the affinity of the whole prion protein for hemin might be greater, with important implications for the interaction occurring in vivo. 

The turbidimetry assay shows that the aggregated hemin-PrP_76–114_ complex is not accumulated in solution over time, but it is followed by a rapid precipitation of the complex. In addition, these data confirm that hemin can increase the propensity of PrP to form insoluble aggregates, even if a more detailed characterization of the secondary structure of the aggregates is required in order to define their toxicity. 

The peroxidase-like reactivity of hemin-PrP complexes, as with other unfolded peptides, exhibits a modest but significant increase with respect to free hemin. The determination of the catalytic parameters shows a small increase for both *k*_1_, that rules the activation of hydrogen peroxide, and *k*_cat_, which is the index of catalytic efficiency. Previous studies from our group indicate that an optimized coordination of the proximal histidine to the iron, which is probably absent in hemin complexes with unstructured peptides, is necessary to strengthen the interaction with the trans-axial ligand (hydrogen peroxide) and maximize the catalytic potential of the complex [63]. However, the binding to PrP peptides potentiates the hemin capability to perform oxidative damage to external substrates, and this may be important in neurodegenerative disorders that occur over a long time span. A situation of particular risk is that associated with traumatic brain injury, where the massive release of hemin becomes the main player in the increase of oxidative stress. 

We strongly believe that the full comprehension at the molecular level of the interaction between neuronal peptides and metal ions plays a key role in understanding the causes and advancement of neurodegenerative diseases and will allow the development of new and effective treatments.

## Figures and Tables

**Table 1:** Equilibrium constants for hemin binding to neuronal peptides in phosphate buffer solution at pH 7.4.

Complex	Log K_1_	Log K_2_	Log β_2_
Hemin-PrP_106–114_	//	//	//
Hemin-PrP_95–114_	4.80 ± 0.06	//	//
Hemin-PrP_84–114_	6.1 ± 0.3	4.72 ± 0.07 ^e^	10.8 ± 0.4 ^e^
Hemin-PrP_76–114_	6.48 ± 0.25	//	//
Hemin-Aβ16 ^a^	4.80 ± 0.02	4.02	8.82 ± 0.02
Hemin-AcR1τ ^b^	6.52 ± 0.02	<2	//
Hemin-Aβ42 ^c^	6.86	6.46	13.32
Hemin-Aβ42 ^d^	6.85	//	//

Data from: ^a^ Thiabaud et al. [42], ^b^ Pirota et al. [43], ^c^ Zhou et al. [44], ^d^ Atamna et al. [45], ^e^ data estimated with two-steps low-affinity equation. // indicates a small value that could not be determined experimentally.

**Table 2:** Kinetic parameters for the catalytic activity of the hemin and hemin-peptide complexes in the oxidation of ABTS by hydrogen peroxide, in phosphate buffer, pH 7.4, at 25 °C.

Complex	*k*_1_ (M^−1^s^−1^)	K_M_ (mM)	*k*_cat_ (s^−1^)	*k*_cat_/K_M_ (M^−1^s^−1^)
Hemin	42 ± 4	1.90 ± 0.20	0.011 ± 0.001	6
Hemin-PrP_106–114_	51 ± 5	4.70 ± 0.40 *	0.068 ± 0.002 *	14 *
Hemin-PrP_95–114_	62 ± 1 *	2.24 ± 0.18	0.048 ± 0.012 *	22 *
Hemin-PrP_84–114_	75 ± 4 *	1.59 ± 0.15	0.054 ± 0.001 *	34 *
Hemin-PrP_76–114_	54 ± 1 *	0.94 ± 0.17 *	0.056 ± 0.002 *	60 *
Hemin-AcR1τ ^a^	73 ± 5	1.90 ± 0.10	0.080 ± 0.002 *	43
Hemin-Aβ16 ^a^	43 ± 5	0.05 ± 0.05	0.070 ± 0.002	134
HRP ^b^	10^7^	0.64	45.5	7.1 × 10^4^

^a^ Data from Pirota et al. [43]. ^b^ Data from Dunford [55]. * parameter significantly different from that of hemin (T-test, *p* = 0.05, *n* = 3).

## Supplementary Materials

Supplementary Materials can be found at https://www.mdpi.com/1422-0067/21/20/7553/s1.

## Abbreviations

PrP	Prion protein
Aβ	amyloid-β
sCJD	sporadic Creutzfeldt–Jakob Disease
OR	octa-repeats
NMDA	*N*-methyl-D-aspartate
PBS	Phosphate-buffered saline
ABTS	2,2′–azino-bis(3-ethylbenzothiazoline-6-sulfonic acid)
TFA	trifluoroacetic acid
DMF	dimethylformamide
Fmoc	fluorenyl methoxycarbonyl
HOBt	N-hydroxybenzotriazole
PyBOP	benzotriazol-1-yl-oxytripyrrolidinophosphonium hexafluorophosphate
DIEA	N,N-diisopropylethylamine
ESI-MS	Electrospray ionization—mass spectrometry
