# Supplementary material for: Interaction between Hemin and Prion Peptides: Binding, Oxidative Reactivity and Aggregation
Source: Int J Mol Sci. 2020 Oct 13;21(20):7553. doi: 10.3390/ijms21207553 (PMC7589926; doi:10.3390/ijms21207553)
Supplement: Supplementary file 1 [file ijms-21-07553-s001.pdf]

Supporting Information

Interaction between hemin and prion peptides: binding, oxidative reactivity and aggregation

Simone Dell'Acqua[1,*], Elisa Massardi[1], Enrico Monzani[1], Giuseppe Di Natale[2], Enrico Rizzarelli[2] and Luigi Casella[1,*]

[1] Dipartimento di Chimica, Università di Pavia, Via Taramelli 12, 27100 Pavia, Italy;

[2] Istituto di Biostrutture e Bioimmagini, Consiglio Nazionale delle Ricerche, Via P. Gaifami 18, Catania, Italy.

* Correspondence: simone.dellacqua@unipv.it; luigi.casella@unipv.it

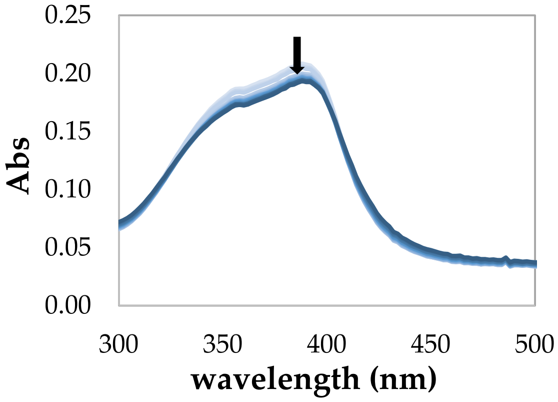

(a)

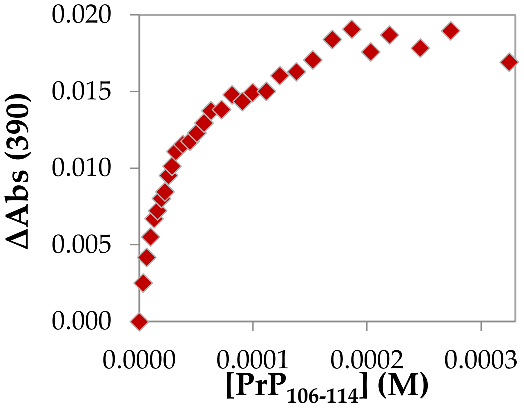

(b)

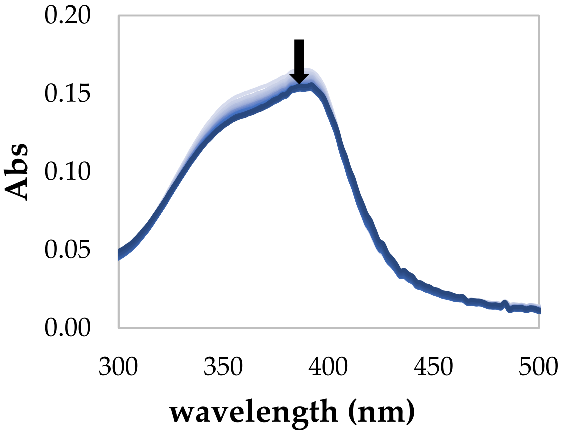

(c)

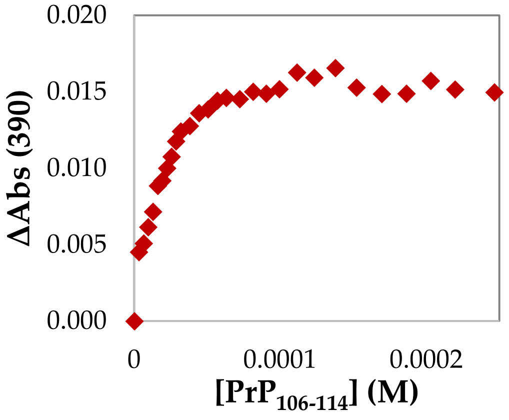

(d)

Figure S1. ***Dataset 1* -** (a) UV-Vis spectrophotometric titration in 20 mM phosphate buffer solution, pH 7.4, showing the decrease in intensity of the Soret band upon binding of $PrP_{106-114}$ (from 0 to 100 equiv., light blue to blue) to hemin (3.23 μM), in a cell of 1 cm path length. The arrow pointing down at 390 nm indicates the hypochromic effect due to the incipient formation of a high-spin hemin-PrP complex. (b) Absorbance changes at 390 nm *vs.* $PrP_{106-114}$ concentration (the sign of subtraction has been inverted in order to obtain positive values). ***Dataset 2* -** (c) UV-Vis spectrophotometric titration in 20 mM phosphate buffer solution, pH 7.4, showing the decrease in intensity of the Soret band upon binding of $PrP_{106-114}$ (from 0 to 100 equiv., light blue to blue) to hemin (2.61 μM), in a cell of 1 cm path length. The arrow pointing down at 390 nm indicates the hypochromic effect due to the

incipient formation of a high-spin hemin-PrP complex. (d) Absorbance changes at 390 nm *vs.* $PrP_{106-114}$ concentration (the sign of subtraction has been inverted in order to obtain positive values).

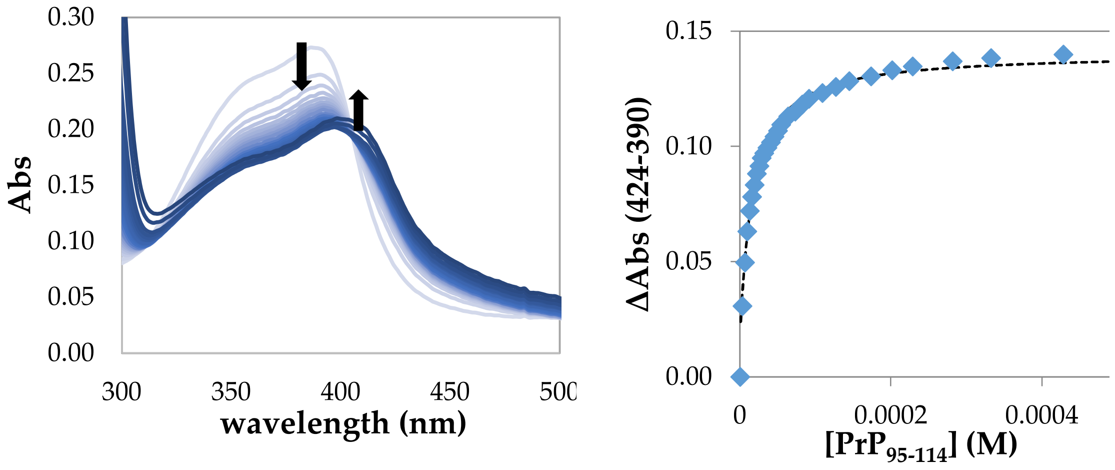

(**a**) (**b**)

Figure S2. (a) UV-Vis spectrophotometric titration (***Dataset 2***) in 20 mM phosphate buffer solution, pH 7.4, showing the decrease in intensity of the Soret band upon binding of hemin (3.29 μM) with PrP_{95-114} (from 0 to 150 equiv., light blue to blue) in a cell of 1 cm path length. The arrow pointing down at 390 nm indicates the hypochromic effect due to the formation of a high-spin hemin-PrP complex; the arrow pointing up at 416 nm indicates the incipient formation of a low-spin complex. (b) Absorbance changes with respect of free hemin at 424 nm with subtraction of the contribution at 390 nm *vs.* PrP_{95-114} concentration fitted by high-affinity equation described in the Materials and Methods section.

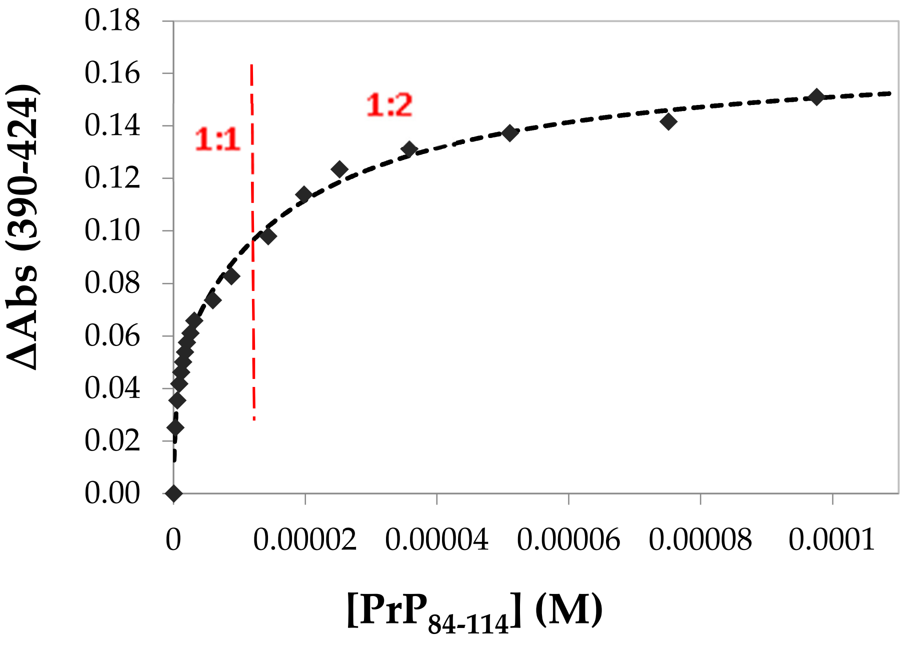

Figure S3. Absorbance changes at 390 nm with subtraction of the contribution at 424 nm vs. PrP84-114 concentration fitted by two-steps low-affinity equation (see Materials and Methods). The red dotted line highlights the two different equilibria.

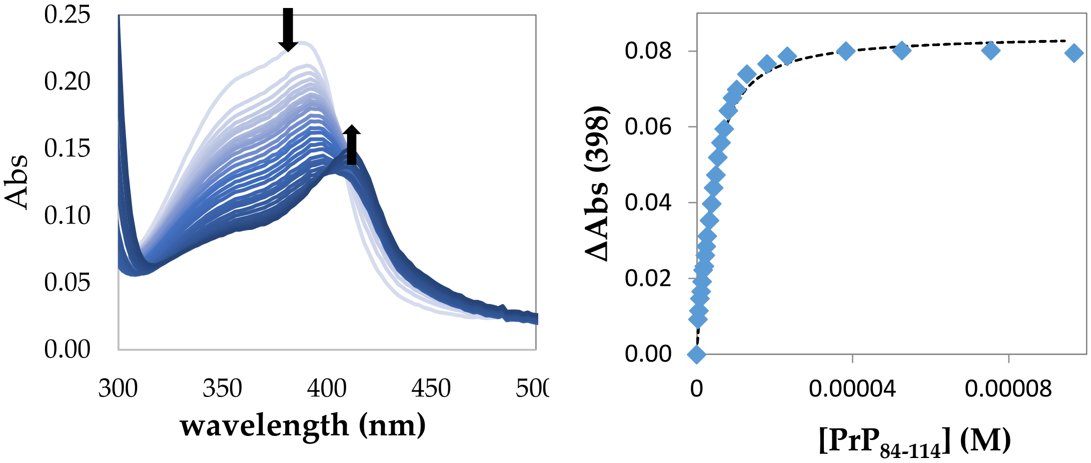

(**a**) (**b**)

Figure S4. (a) - UV-Vis spectrophotometric titration (***Dataset 2***) in 20 mM phosphate buffer solution, pH 7.4, showing the decrease in intensity of the Soret band upon binding of hemin (2.80 μM) with PrP_{84-114} (from 0 to 40 equiv., light blue to blue) in a cell of 1 cm path length. The arrow pointing down at 390 nm indicates the hypochromic effect due to the formation of a high-spin hemin-PrP complex; the arrow pointing up at 416 nm indicates the incipient formation of a low-spin complex. (b) Absorbance changes with respect of free hemin at 398 nm *vs.* PrP_{84-114} concentration fitted by high-affinity equation described in the Materials and Methods section (the sign of ΔAbs (398) has been changed in order to obtain positive values).

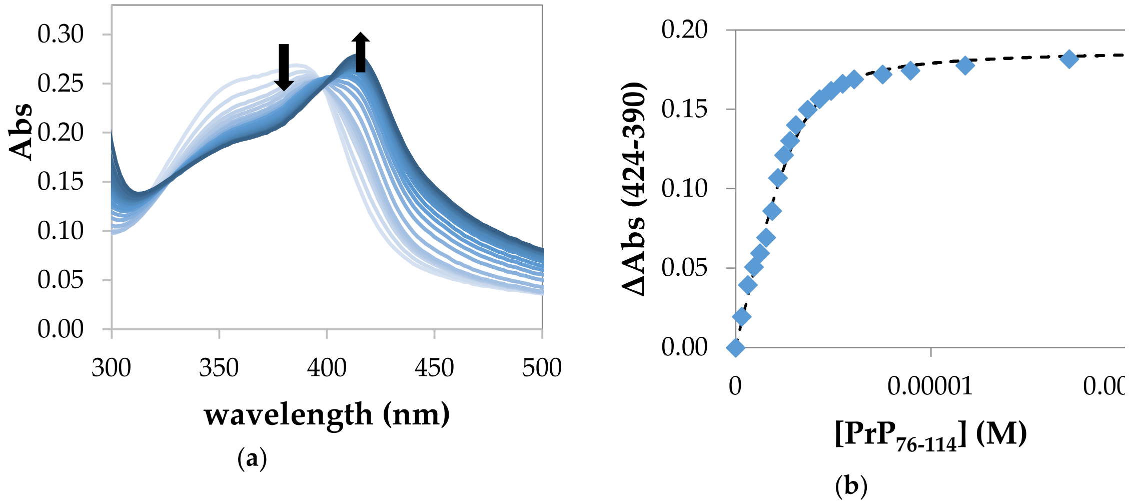

(**a**) (**b**)

Figure S5. (a) - UV-Vis spectrophotometric titration (***Dataset 2***) in 20 mM phosphate buffer solution, pH 7.4, showing the decrease in intensity of the Soret band upon binding of hemin (2.74 μM) with PrP_{76-114} (from 0 to 6 equiv., light blue to blue) in a cell of 1 cm path length. The arrow pointing down at 390 nm indicates the hypochromic effect due to the formation of a high-spin hemin-PrP complex; the arrow pointing up at 416 nm indicates the formation of a low-spin complex. (b) Absorbance changes with respect of free hemin at 424 nm with subtraction of the contribution at 390 nm *vs.* PrP_{76-114} concentration fitted by high-affinity equation described in the Materials and Methods section.

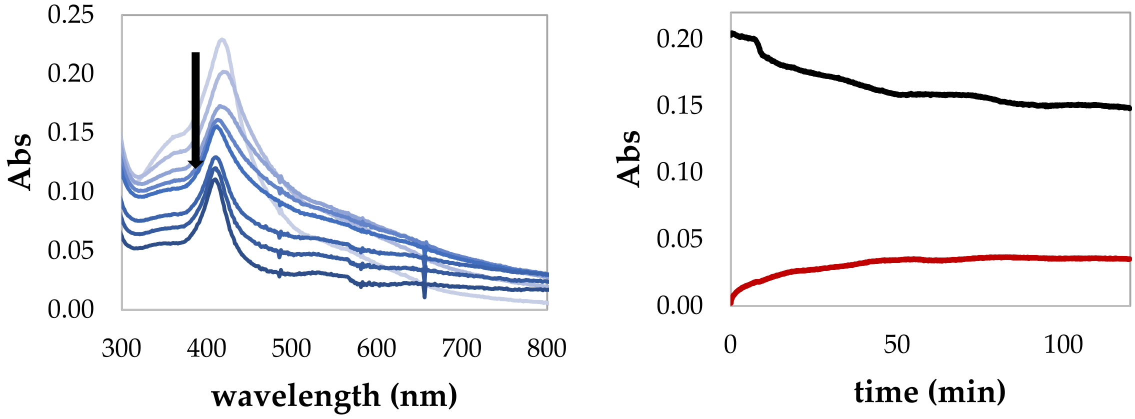

(**b**)

(**a**)

Figure S6. PrP$_{76-114}$ (8 μM) aggregation and precipitation mediated by hemin (4 μM), monitored by UV-visible spectroscopy at pH 7.4 in PBS: (a) UV-visible spectra taken at 1, 15, 45, 90, 120, 330, 480 and 840 minutes (from light blue to blue); (b) absorbance changes at 405 nm (black trace) and 750 nm (red trace) *vs.* time.

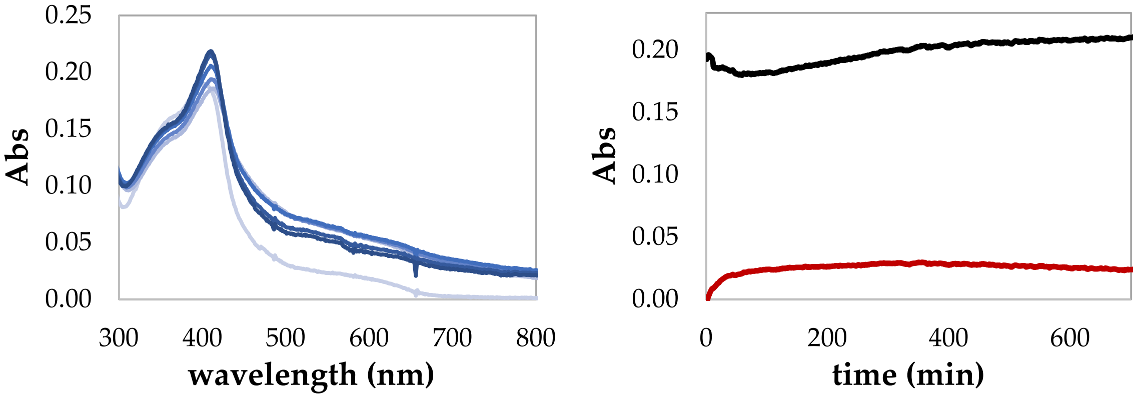

(**b**)

(**a**)

Figure S7. PrP$_{84-114}$ (8 μM) aggregation and precipitation mediated by hemin (4 μM), monitored by UV-visible spectroscopy at pH 7.4 in PBS: (a) UV-visible spectra taken at 1, 60, 150, 240, 450, 600 and 900 minutes (from light blue to blue); (b) absorbance changes at 405 nm (black trace) and 750 nm (red trace) *vs.* time.

Kinetic experiments

Plots corresponding to the determination of the rate dependence on the concentration of hydrogen peroxide (Figure S5), at fixed substrate (ABTS) concentration, and the rate dependence on substrate concentration (Figure S6), at saturating H_2O_2 concentration, for the oxidation of ABTS by hydrogen peroxide catalyzed by the hemin and hemin-PrP peptide complexes.

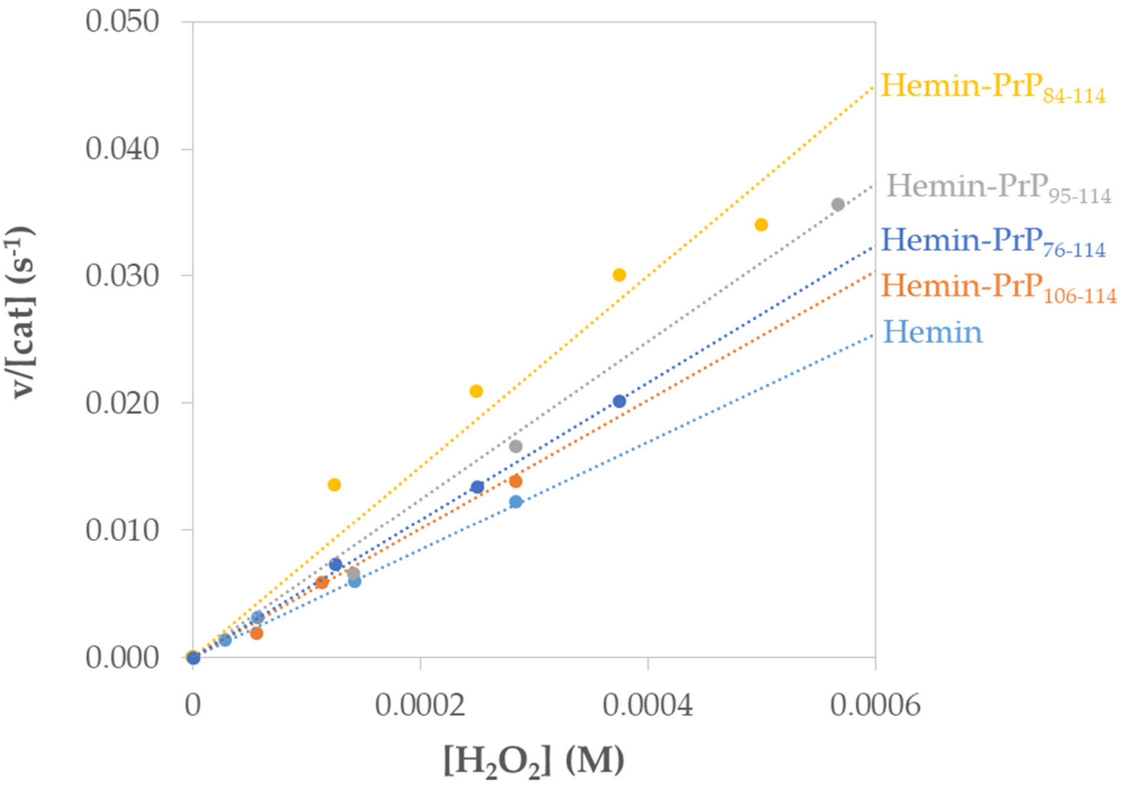

Figure S8. Determination of the H_2O_2 concentration dependence of the reaction rate. Concentration of the hemin catalysts was 3 µM in all cases. The 1:1 hemin-peptide complexes were generated by adding 90 µM PrP106-114, 4.8 µM PrP95-114, 6 µM PrP84-114 and 3.6 µM PrP76-114 to the hemin solution prior to starting the experiments. The concentration of ABTS was maintained fixed at 1 mM. The experiments were carried out in phosphate buffer 20 mM at pH 7.4 at 25 °C. The reaction rates (in s^{-1}) were obtained by dividing the initial rates for the catalyst concentration, the optical path (1 cm), the extinction coefficient of the product $ABTS^{+}$ (ε_{660} = 14700 M^{-1} cm^{-1}) and a factor 2 because one cycle corresponds to two $ABTS^{+}$ formed.[62].

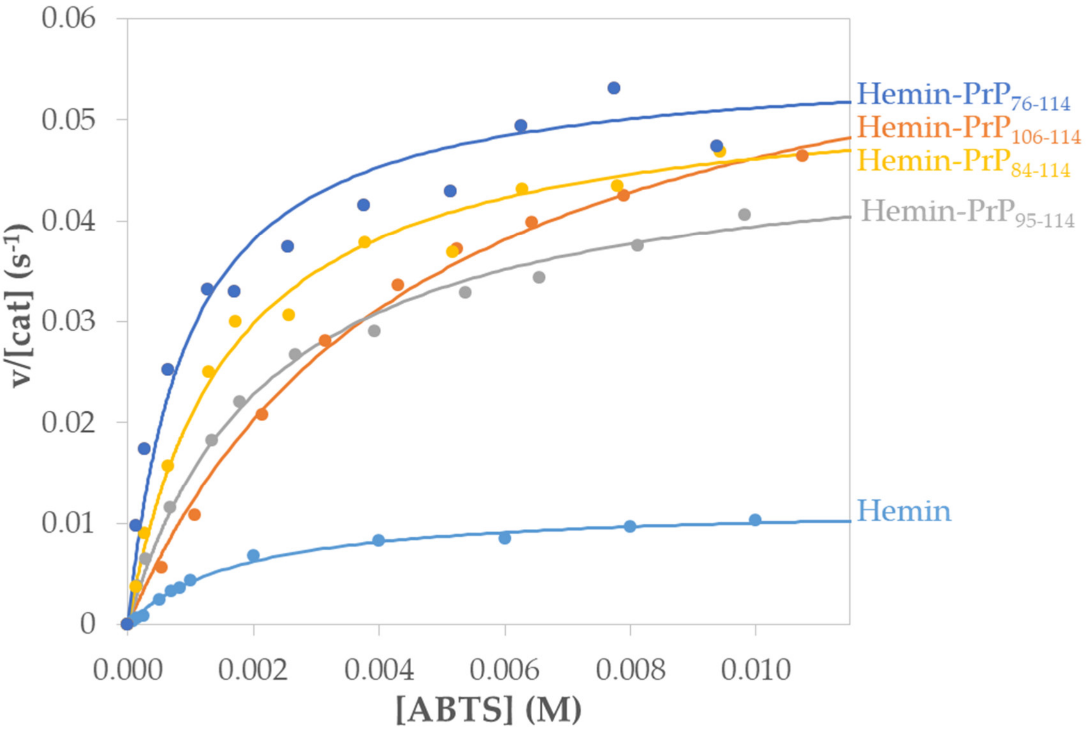

Figure S9. Determination of the substrate concentration dependence of the reaction rate. Plots of the initial rates *vs.* ABTS concentration for $ABTS^{\cdot+}$ formation catalyzed by the various hemin complexes (3 μM). The catalysts are: hemin-$PrP_{106-114}$, hemin-PrP_{95-114}, hemin-PrP_{84-114}, hemin-PrP_{76-114}, and free hemin. The hemin-peptide complexes were obtained by adding 90 μM $PrP_{106-114}$, 4.8 μM PrP_{95-114}, 6 μM PrP_{84-114} and 3.6 μM PrP_{76-114} and hydrogen peroxide concentrations to the hemin solution as above. The concentration of H_2O_2 was saturating in all cases (5 mM for hemin, and 11 mM $PrP_{106-114}$, 10 mM PrP_{95-114}, 8 mM PrP_{84-114} and 8 mM PrP_{76-114} for hemin-peptide complexes). The experiments were carried out at 25 °C in 20 mM phosphate buffer, pH 7.4. The reaction rates were determined as above.
